# Serum Legumain Is Associated with Peripheral Artery Disease in Patients with Type 2 Diabetes

**DOI:** 10.1155/2021/5651469

**Published:** 2021-12-18

**Authors:** Wen Wei, Shujin Chen, Jianqing Huang, Yan Tong, Jushun Zhang, Xiuping Qiu, Wenrui Zhang, Hangju Chen, Rong Huang, Jin Cai, Mei Tu

**Affiliations:** ^1^Department of Endocrinology, Fujian Longyan First Hospital, Longyan First Affiliated Hospital of Fujian Medical University, Longyan 364000, China; ^2^The Second School of Clinical Medicine, Southern Medical University, Guangzhou 510515, China; ^3^Department of Ultrasonography, Fujian Longyan First Hospital, Longyan First Affiliated Hospital of Fujian Medical University, Longyan 364000, China; ^4^Department of Neurosurgery, Ren Ji Hospital, School of Medicine, Shanghai Jiaotong University, Shanghai 200240, China; ^5^Department of Endocrinology, Fujian Longyan First Hospital, Fujian Medical University, Fuzhou 350004, China

## Abstract

**Background:**

Legumain is related to carotid atherosclerotic plaques and may be a new biomarker of carotid atherosclerosis. However, the association between legumain and peripheral artery disease (PAD) of lower extremity has been less studied. This study is aimed at exploring the potential link between legumain and PAD in patients with type 2 diabetes mellitus (T2DM).

**Methods:**

A cross-sectional study was conducted on 483 hospitalized T2DM patients. The serum legumain level was measured by a sandwich enzyme-linked immunosorbent assay. PAD was evaluated by color Doppler sonography. The association between legumain and PAD was tested by logistic regression. The predictive power of legumain for PAD was evaluated with the receiver-operating-characteristic (ROC) curve.

**Results:**

Overall, 201 (41.6%) patients suffered from PAD. Patients with PAD had significantly higher serum legumain level than those without PAD [11.9 (6.3, 17.9) *μ*g/L vs. 7.6 (3.2, 14.2) *μ*g/L, *p* < 0.001]. Logistic regression showed that a higher serum legumain level was independently associated with a greater risk of PAD in T2DM patients [adjusted odds ratio (aOR): 1.03; 95% confidence interval (CI): 1.01-1.06]. The area under the ROC curve was 0.634 (95% CI, 0.585 to 0.684).

**Conclusion:**

High serum legumain level was significantly correlated with an increased risk of PAD in T2DM patients.

## 1. Introduction

Peripheral artery disease (PAD) of the lower extremity is one of the common macrovascular complications of diabetes and is associated with a substantial increase in the risk of foot ulceration and amputation [1, 2]. Furthermore, PAD is an independent predictor of cardiovascular and cerebrovascular ischemic events, the leading causes of mortality and morbidity in diabetic patients [3]. Early detection and treatment of PAD are critical to prevent amputation and mortality of the diabetic population.

Legumain, also known as asparaginyl endopeptidase (AEP), belongs to the C13 family of cysteine proteases that include caspases and separases [4]. Legumain has been found to play a role in antigen presentation [5], invasion/metastasis of tumor [6, 7], and neurodegenerative diseases [8–10]. Recent studies indicate that legumain is upregulated in carotid atherosclerotic plaques and might be a new and early biomarker of carotid atherosclerosis [11–13]. However, no study has evaluated the serum legumain level in patients with PAD of the lower extremity.

Therefore, in this study, we aim to evaluate the association between serum legumain and PAD and whether serum legumain could be used as a predictor of PAD in patients with type 2 diabetes mellitus (T2DM).

## 2. Methods

### 2.1. Study Population and Data Sources

The cross-sectional study included adult patients (≥18 years of age) with T2DM who were hospitalized at Longyan First Affiliated Hospital of Fujian Medical University, Fujian, China, between July 2018 and June 2019. T2DM was diagnosed according to the World Health Organization (WHO) criteria. Patients with other types of diabetes and pregnant women were excluded. We also excluded patients with ketoacidosis, hyperosmolar status, acute severe infection, chronic or acute renal diseases on hemodialysis, autoimmune disease, malignant cancer, and severe cardiac insufficiency. Eventually, 483 patients were included. The study was approved by the institutional Ethics Research Committee of Longyan First Affiliated Hospital of Fujian Medical University. All patients gave written informed consent for participation in the study.

Data were extracted from the electronic clinical management records system of Longyan First Affiliated Hospital of Fujian Medical University. Information mainly included demographic characteristics, medical history, medications, laboratory test results, and other clinical variables. Height, weight, waist circumference (WC), and blood pressure (BP) were assessed on a standardized form by the nurse on admission. Venous blood samples were collected in the early morning after overnight fasting.

Body mass index (BMI) was calculated as weight/height^2^ (kg/m^2^). Homeostatic model assessment-insulin resistance (HOMA-IR) was calculated using the following formula: fasting blood glucose (mmol/L) × fasting plasma insulin (mU/L)/22.5. Estimated glomerular filtration rate (eGFR) value was calculated based on serum creatinine (Scr) level using the Modification of Diet in Renal Disease (MDRD) formula.

### 2.2. Ultrasonography Measurements

We followed the methods of He et al. [14]. Color Doppler sonography was carried out with a Philips Ultrasound (Epiq5, Bothell, WA) equipped with a 5-12 MHz linear array transducer. The ultrasonography was conducted by two experienced ultrasonographers according to a standardized technique. After the participants had kept in the supine position for 5 min, the transducer was placed on the lower limbs to show both vessel imaging and blood flow characteristics. Lower extremity arteries were evaluated bilaterally at the levels of the seven locations: common femoral artery, profunda femoris artery, superficial femoral artery, popliteal artery, anterior tibial artery, posterior tibial artery, and peroneal artery. At each location, intima-media thickness (IMT) and atherosclerotic plaques were recorded. The IMT on both sides was measured as the distance between the leading edge of the lumen-intima echo and the leading edge of the media-adventitia echo [15].

Artery intimal thickening was defined as a focal thickening from the intima-lumen interface to the media-adventitia interface of over 1.0 mm. Atherosclerotic plaque was defined as a focal thickening of the intima-media complex encroaching into the arterial lumen by at least 0.5 mm or involving 50% of the surrounding IMT or a focal thickening from the intima-lumen interface to the media-adventitia interface of over 1.5 mm [16]. PAD was defined as the presence of arterial atherosclerotic plaques in any of the aforementioned arterial segments [17, 18].

### 2.3. Measurement of Legumain

After a fast for 8 hours, approximately 2-3 mL of peripheral blood was obtained using a collection tube without additives from each subject within 24 hours after admission. Serum legumain (*μ*g/L) concentration was measured using a commercially available enzyme-linked immunosorbent assay (Jianglai Biotechnology Co., Ltd., Shanghai, China) according to the manufacturer's instructions.

### 2.4. Bias Control

A number of measures were taken to mitigate bias, such as strict inclusion and exclusion criteria, standardized measurement procedures for anthropometric indexes and blood indicators, measurement of arterial plaque according to a standardized technique by two experienced ultrasonographers, and a multivariable regression model to correct confounding factors in statistical analysis.

### 2.5. Sample Size Consideration

To obtain unbiased estimates of the regression coefficients, it has previously been suggested to include 10-20 events per degree of freedom in the predictors of logistic regression analysis. The study consisted of 483 patients, 201 (41.6%) of whom had PAD. Thus, the event per variable (EPV) value was 10-20 for the regression model.

### 2.6. Statistical Analyses

Continuous variables were expressed as mean ± standard deviation or median (quartile range), and categorical variables were expressed as frequency counts and percentages. Baseline characteristics stratified by whether or not PAD were compared using the two-sample independent *t*-test or the Mann-Whitney *U* nonparametric test for continuous variables and the chi-square test for categorical variables. The trend of PAD prevalence with serum legumain level changes (cut-off by quartile range) was assessed using the Cochran-Armitage test.

The correlation between PAD and legumain was tested by univariable and multivariable logistic regression analyses. Variables that were entered into the multivariable model were carefully selected based on variables associated with known risk factors or variables with *p* value < 0.05 in baseline or in univariable regression analysis. The predictive power of legumain for PAD was evaluated with the receiver-operating-characteristic (ROC) curve using DeLong's method and expressed by the C-statistic.

Considering that legumain is upregulated in carotid atherosclerotic plaques and that most PAD patients likely have concomitant carotid plaques, we performed sensitivity analysis in T2DM patients without carotid plaque. There was a very small amount of missing data for variables of interest, so we did not deal with missing values (Supplementary Table 3).

All analyses were performed with R software (version 4.0.5; R Foundation for Statistical Computing, Vienna, Austria). A two-sided *p* value < 0.05 indicated significance for all analyses.

## 3. Results

### 3.1. Clinical Characteristics

A total of 483 T2DM patients with the mean age of 58 ± 11 years, including 284 men and 199 women, were included in this study. The prevalence of PAD was 41.6%. The patients were divided into two groups based on the presence of PAD. The clinical characteristics of the subjects are summarized in Table 1.

Compared with patients without PAD, patients with PAD were likely to be older (62 ± 10 years vs. 54 ± 10 years, *p* < 0.001) and be male (64.7% vs. 54.6%, *p* = 0.034). These patients demonstrated a higher legumain level [11.9 (6.3, 17.9) *μ*g/L vs. 7.6 (3.2, 14.2) *μ*g/L, *p* < 0.001], homocysteine (HCY) level (11.5 ± 3.1 vs. 10.6 ± 3.5 *μ*mol/L, *p* = 0.006), and HOMA-IR value [17.3 (8.1, 31.9) vs. 12.9 (7.2, 26.1), *p* = 0.037]. They also had longer duration of diabetes [8 (3, 11) years vs. 5 (1, 10) years, *p* < 0.001], accompanied with higher rates of diabetic complications such as diabetic retinopathy (DR) (29.4% vs. 16.3%, *p* = 0.009), diabetic peripheral neuropathy (DPN) (67.2% vs. 51.6%, *p* = 0.023), and diabetic nephropathy (DN) (26.6% vs. 10.4%, *p* < 0.001) (Table 1).

The incidences of smoking, hypertension, coronary artery disease (CAD), and stroke in patients with PAD were higher than those in patients without (43.8% vs. 28.7%, *p* = 0.001; 51.2% vs. 34.4%, *p* < 0.001; 13.9% vs. 2.5%, *p* < 0.001; 7.0% vs. 1.4%, *p* = 0.003, respectively). Medication use is also shown in Table 1.

### 3.2. Association between Serum Legumain Level and PAD

**Figure 1:**
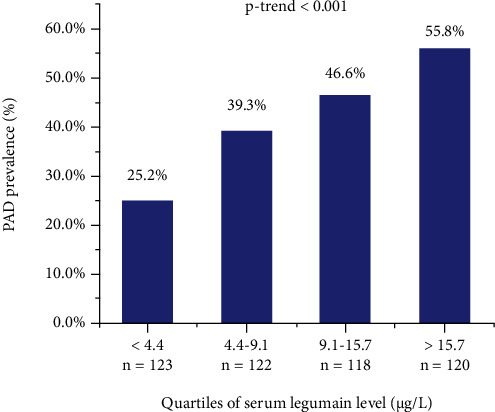
Prevalence of PAD stratified by quartiles of serum legumain level.

The median (25th and 75th percentiles) serum legumain level among the entire study population was 9.1 (4.4-15.7) *μ*g/L. The prevalence of PAD showed an increasing trend in higher serum legumain levels (Figure 1).

Multivariable logistic regression showed that the higher serum legumain level was independently associated with a greater risk of PAD in T2DM patients (adjusted odds ratio [aOR]: 1.03; 95% confidence interval [CI]:1.01-1.06). The covariates included in the multivariable analysis were age, gender, duration of diabetes, hypertension, low-density lipoprotein cholesterol (LDL-C), hypersensitive C-reactive protein (hs-CRP), HCY, HOMA-IR, eGFR, smoke, DR, and carotid atherosclerotic plaque (Table 2).

**Figure 2:**
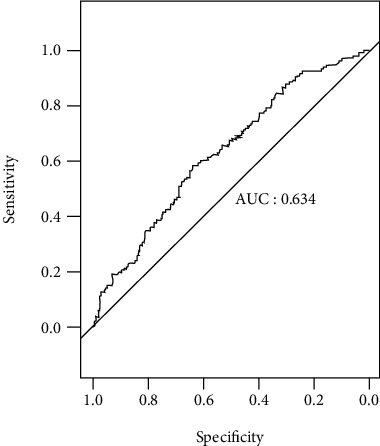
Receiver operator characteristic (ROC) curve analysis.

The area under the ROC curve was 0.634 (95% confidence interval [CI], 0.585 to 0.684). At a cutoff of 10.2 *μ*g/L, serum legumain exhibited a sensitivity of 58.7% and a specificity of 64.2% for detecting PAD (Figure 2).

### 3.3. Sensitivity Analyses

A total of 200 diabetic patients without carotid plaque were included in sensitivity analyses. The prevalence of PAD was 23.5%. Multivariable logistic regression showed that a higher serum legumain level was independently associated with a greater risk of PAD (aOR: 1.05; 95% CI: 1.02-1.10) (Supplementary Table 1-2).

## 4. Discussion

The present study was the first to explore the relationship between serum legumain and PAD in patients with T2DM. The results demonstrated that patients with PAD showed a higher serum legumain level than those without, and the prevalence of PAD increased with the increase of the serum legumain level. A high legumain level was independently associated with a greater risk of PAD.

PAD is one of the common macrovascular complications of diabetes. Diabetes was significantly related with the presence of atherosclerotic plaques in the lower extremity [19]. The prevalence of atherosclerotic plaques in femoral arteries was 77% in elderly Finns with diabetes or impaired glucose tolerance [20]. The prevalence of lower extremity artery plaques in our study was 41.6%, but the patients in our study were younger with the mean age of 58 years. Diabetes-associated PAD is often associated with diabetic peripheral neuropathy [21]. Decreased pain and body temperature caused by neuropathy may mask the symptoms of PAD (such as resting pain), leading to delayed diagnosis of PAD and more serious consequences such as gangrene and amputations [22, 23]. Due to the similar pathophysiological mechanisms (such as oxidative stress and endothelial dysfunction) of PAD and atherosclerosis in other vessels, PAD is closely related to the occurrence of cardiovascular disease [1, 2, 24]. Given the heavy public health burden, economic burden, and poor prognosis caused by diabetes-related amputations [25], early identification of effective biomarkers for PAD is essential. Our study showed that the high serum legumain level significantly correlated with an increased risk of PAD in T2DM patients. Measurement of the serum legumain level may allow clinicians to identify diabetic patients at elevated risk for PAD. However, the predictive value of the serum legumain for PAD needs to be confirmed in further larger prospective studies.

It is known that atherosclerosis is characterized by a complex process of vascular injury; inflammation, with monocyte adhesion to endothelial cells (ECs); lipid deposition within macrophage foam cells; neointimal hyperplasia, involving vascular smooth muscle cells (VSMCs); and extracellular matrix (ECM) remodeling [26, 27]. The mechanism underlying the relationship between legumain and arterial atherosclerotic plaque may be as follows. Clerin et al. indicated that the increased legumain level correlated with increased accumulation of inflammatory cells in early- to late-stage atherosclerotic lesions in both human coronary arteries and mouse aortas. Legumain gene expression could be regulated by proinflammatory cytokines such as interleukin-1*β* (IL-1*β*), interferon-*γ* (IFN-*γ*), or tumor necrosis factor-*α* (TNF-*α*) [28]. Legumain promoted atherosclerotic vascular remodeling by enhancing macrophage foam cell formation; VSMC migration; and collagen-3, fibronectin, and elastin production by VSMCs [29]. Papaspyridonos et al. demonstrated that legumain was coexpressed with matrix metalloproteinases (MMPs), so it was regarded as a contributor to plaque rupture [30]. Legumain has also been reported to facilitate atherosclerotic plaque formation and plaque rupture via enhanced ECM degradation [11].

Consistent with previous studies, we found that some of the traditional risk factors for atherosclerosis were also present in this population. As expected, age, smoking, hypertension, duration of diabetes, and insulin resistance were independently associated with the presence of PAD in the T2DM patients. Therefore, smoking cessation and treatment of hypertension and insulin resistance are important in order to prevent atherosclerosis in the lower extremity arteries in diabetic patients. The association between legumain and inflammation has been indicated elsewhere [12, 31]. Chronic inflammation has been established as a major mechanism to cause insulin resistance and atherosclerosis [32]. Therefore, the serum legumain level may reflect the systemic inflammation in patients with atherosclerosis. Serum legumain associated with atherosclerosis and inflammation may improve risk stratification in T2DM patients. On the other hand, the benefit of targeting legumain in atherosclerosis therapy may be its effect on inflammation.

In diabetic patients, atherosclerotic lesions were more common in lower extremity arteries than carotid arteries. Schneider et al. found that PAD but not carotid and/or coronary artery diseases was a relevant risk factor for both minor and major amputation [33]. Furthermore, PAD was closely associated with the occurrence of cardiovascular disease. The latest researches demonstrated that legumain was associated with the presence of complex coronary lesions and the outcome in acute cardiovascular events [31, 34–36]. Therefore, a follow-up of the outcome in PAD subjects is necessary to elucidate whether serum legumain might be a clinically relevant vascular disease biomarker or a new strategy for PAD.

Our study has several limitations. First, the cross-sectional design limits our ability to assess the causal relationship between serum legumain and PAD, and the predictive value of serum legumain for PAD needs to be confirmed in further larger prospective studies that include a nondiabetic population. Second, the ankle-brachial index and symptoms of intermittent claudication or ischemic resting pains were not detected in this study. Third, the precise regulatory mechanism of legumain and atherosclerotic plaque needs further investigation. Finally, it was a cross-sectional single-center study with some inherent bias. However, efforts were made to mitigate bias.

## 5. Conclusion

In conclusion, the high serum legumain level was independently associated with an increased risk of PAD in Chinese patients with T2DM. Measurement of the serum legumain level may allow clinicians to identify diabetic patients at elevated risk for PAD. Further studies revealing the immanent connection of legumain with the pathology of diabetes-associated PAD may confirm the predictive value of serum legumain for PAD and provide a new strategy for PAD.

## Figures and Tables

**Table 1:** Comparison of clinical characteristics between patients with and without PAD.

Characteristic	With PAD (*n* = 201)	Without PAD (*n* = 282)	*p* value
Demographic characteristics			
Age (years)	62 ± 10	54 ± 10	<0.001
Male, *n* (%)	130 (64.7)	154 (54.6)	0.034
WC (cm)	90.1 ± 9.0	89.1 ± 10.3	0.335
BMI (kg/m^2^)	24.0 ± 2.8	24.5 ± 3.7	0.111
Medical history and clinical condition			
Smoking history, *n* (%)	88 (43.8)	81 (28.7)	0.001
Hypertension, *n* (%)	103 (51.2)	97 (34.4)	<0.001
SBP (mmHg)	140.7 ± 19.2	134.6 ± 18.3	<0.001
DBP (mmHg)	81.8 ± 12.7	82.7 ± 12.0	0.436
Duration of diabetes (years)	8 (3, 11)	5 (1, 10)	<0.001
DR, *n* (%)	45 (29.4)	34 (16.3)	0.009
DPN, *n* (%)	88 (67.2)	83 (51.6)	0.023
DN, *n* (%)	41 (26.6)	21 (10.4)	<0.001
Stroke, *n* (%)	14 (7.0)	4 (1.4)	0.003
NAFLD, *n* (%)	91 (54.2)	134 (54.9)	0.960
Carotid plaque, *n* (%)	149 (76.0)	121 (44.2)	<0.001
Laboratory examination			
Legumain (*μ*g/L)	11.9 (6.3, 17.9)	7.6 (3.2, 14.2)	<0.001
FBG(mmol/L)	9.2 ± 3.4	8.8 ± 3.4	0.242
2hPBG (mmol/L)	13.0 ± 4.8	13.0 ± 4.9	0.965
HbA1C (%)	10.4 ± 2.4	10.4 ± 2.7	0.945
HOMA-IR	17.3 (8.1, 31.9)	12.9 (7.2, 26.1)	0.037
TG (mmol/L)	2.1 ± 1.7	2.0 ± 1.6	0.513
TC (mmol/L)	4.9 ± 1.3	4.9 ± 1.2	0.831
HDL-C (mmol/L)	1.3 ± 0.6	1.3 ± 0.4	0.357
LDL-C (mmol/L)	3.1 ± 1.1	3.2 ± 1.0	0.748
eGFR (mL/min/1.73mm^2^)	107.6 ± 37.7	118.5 ± 37.3	0.003
UA (*μ*mol/L)	350.9 ± 95.0	341.3 ± 89.3	0.292
hs-CRP (mg/L)	1.4 (0.7, 3.0)	1.2 (0.6, 2.2)	0.091
HCY (*μ*mol/L)	11.5 ± 3.1	10.6 ± 3.5	0.006
Administered drugs			
Insulin, *n* (%)	46 (22.9)	47 (16.7)	0.111
OADs, *n* (%)	129 (64.5)	160 (57.3)	0.138
Statins, *n* (%)	18 (9.0)	6 (2.1)	0.001
Aspirin, *n* (%)	17 (8.5)	7 (2.5)	0.006
ACEI/ARB, *n* (%)	36 (18.0)	27 (9.7)	0.012
CCB, *n* (%)	42 (21.0)	33 (11.8)	0.009
*β*-Blockers, *n* (%)	15 (7.5)	8 (2.9)	0.034
Diuretic, *n* (%)	6 (3.0)	5 (1.8)	0.575

Abbreviation: PAD: peripheral artery disease; WC: waist circumference; BMI: body mass index; SBP: systolic blood pressure; DBP: diastolic blood pressure; DR: diabetic retinopathy; DPN: diabetic peripheral neuropathy; DN: diabetic nephropathy; NAFLD: nonalcoholic fatty liver disease; FBG: fasting blood glucose; 2hPBG: 2 hours postprandial blood glucose; HbA1c: glycosylated hemoglobin; HOMA-IR: homeostatic model assessment-insulin resistance; TG: triglyceride; TC: total cholesterol; HDL-C: high-density lipoprotein cholesterol; LDL-C: low-density lipoprotein cholesterol; eGFR: estimated glomerular filtration rate; UA: uric acid; hs-CRP: hypersensitive C-reactive protein; HCY: homocysteine; OADs: oral antidiabetic drugs; ACEI/ARB: angiotensin-converting enzyme inhibitor/angiotensin receptor blocker; CCB: calcium channel blocker.

**Table 2:** Logistic regression analysis for PAD.

	Univariable		Multivariable	
	OR (95% CI)	*p* value	aOR (95% CI)	*p* value
Legumain	1.02 (1.01-1.03)	0.001	1.03 (1.01-1.06)	0.006
Age	1.08 (1.06-1.10)	<0.001	1.12 (1.07-1.17)	<0.001
Male	1.52 (1.05-2.21)	0.027	1.74 (0.71-4.29)	0.224
Duration of diabetes	1.08 (1.04-1.11)	<0.001	1.09 (1.02-1.16)	0.007
Hypertension	2.01 (1.39-2.91)	<0.001	2.77 (1.40-5.60)	0.004
LDL-C	0.97 (0.81-1.17)	0.748	1.21 (0.86-1.73)	0.275
hs-CRP	1.06 (1.02-1.12)	0.009	0.96 (0.86-1.07)	0.484
HCY	1.08 (1.02-1.14)	0.007	0.95 (0.85-1.06)	0.352
HOMA-IR	1.01 (1.00-1.02)	0.005	1.02 (1.01-1.04)	0.009
eGFR	0.99 (0.99-1.00)	0.004	1.01 (1.00-1.02)	0.208
Smoking history	1.93 (1.32-2.83)	0.001	3.53 (1.40-9.34)	0.009
DR	1.84 (1.20-2.86)	0.006	1.02 (0.51-2.01)	0.954
Carotid plaque	4.01 (2.69-6.06)	<0.001	1.29 (0.63-2.65)	0.487
BMI	0.96 (0.90-1.01)	0.113		
WC	1.01 (0.99-1.03)	0.334		
SBP	1.02 (1.01-1.03)	0.001		
DPN	1.42 (0.96-2.10)	0.082		
DN	1.39 (0.98-1.97)	0.063		
Stroke	5.20 (1.83-18.57)	0.004		
FBG	1.03 (0.98-1.09)	0.242		
2hPBG	1.00 (0.96-1.04)	0.965		
HbA1C	1.00 (0.93-1.08)	0.945		
TG	1.04 (0.92-1.17)	0.513		
TC	0.98 (0.84-1.15)	0.831		
HDL-C	1.21 (0.80-1.88)	0.362		
UA	1.00 (1.00-1.00)	0.292		
Insulin	1.48 (0.94-2.34)	0.089		
OADs	1.35 (0.93-1.97)	0.115		
Statins	4.53 (1.86-12.67)	0.002		
Aspirin	3.63 (1.53-9.55)	0.005		

Abbreviation as in Table 1.

## Data Availability

The data used to support the findings of this study have not been made available because of patient privacy.

## Supplementary Materials

Supplementary Materials The sensitivity analysis in T2DM patients without carotid plaque and the information of missing values of baseline variables in total patients were included in supplementary materials.
