# Supplementary material for: Serum Legumain Is Associated with Peripheral Artery Disease in Patients with Type 2 Diabetes
Source: J Diabetes Res. 2021 Dec 18;2021:5651469. doi: 10.1155/2021/5651469 (PMC8710170; doi:10.1155/2021/5651469)
Supplement: Supplementary Materials — The sensitivity analysis in T2DM patients without carotid plaque and the information of missing values of baseline variables in total patients were included in supplementary materials. [file 5651469.f1.docx]

**Supplementary Table 1.** **Baseline characteristics of patients without carotid plaque by PAD groups**

| **Characteristic** | **With PAD**  **（n=47）** | **Without PAD**  **（n=153）** | **p-value** |
| --- | --- | --- | --- |
| **Demographic characteristics** | | | |
| age (years) | 55 ± 8 | 51 ± 10 | 0.024 |
| male, n (%) | 32 (68.1) | 87 (56.9) | 0.230 |
| WC (cm) | 90.9 ± 9.7 | 89.6 ± 11.3 | 0.546 |
| BMI (kg/m^2^) | 24.6 ± 2.6 | 24.7 ± 3.9 | 0.885 |
| **Medical history and Clinical condition** | | | |
| smoking history, n (%) | 21 (44.7) | 43 (28.1) | 0.051 |
| hypertension, n (%) | 23 (48.9) | 46 (30.1) | 0.027 |
| SBP (mmHg) | 140.9 ± 17.9 | 132.8 ±18.4 | 0.009 |
| DBP (mmHg) | 87.1 ± 11.5 | 83.6 ± 12.3 | 0.088 |
| duration of diabetes (years) | 6 (2, 10) | 4 (0, 10) | 0.222 |
| DR, n (%) | 5 (14.3) | 16 (14.3) | 0.998 |
| DPN, n (%) | 17 (58.6) | 50 (58.8) | 0.649 |
| DN, n (%) | 7 (20.0) | 7 (6.4) | 0.045 |
| stroke, n (%) | 3 (6.4) | 1 (0.7) | 0.063 |
| NAFLD, n (%) | 25(61.0) | 80 (58.4) | 0.909 |
| **Laboratory examination** | | | |
| legumain (μg/L) | 8.2(3.9, 16.2) | 6.7 (2.5, 11.4) | 0.080 |
| FBG(mmol/L) | 9.1 ± 3.6 | 9.1 ± 3.4 | 0.958 |
| 2hPBG(mmol/L) | 12.9 ± 5.1 | 13.2 ± 4.8 | 0.738 |
| HbA1C (%) | 10.2 ± 2.7 | 10.2 ± 2.5 | 0.920 |
| HOMA-IR | 15.3 (9.0, 31.2) | 14.7(7.3, 27.9) | 0.482 |
| TG (mmol/L) | 2.4 ± 2.3 | 2.1 ± 1.6 | 0.278 |
| TC (mmol/L) | 4.9 ± 1.2 | 5.1 ± 1.2 | 0.413 |
| HDL-C(mmol/L) | 1.3 ± 0.4 | 1.3 ± 0.4 | 0.810 |
| LDL-C(mmol/L) | 3.1 ± 1.0 | 3.3 ± 1.0 | 0.326 |
| eGFR(ml/min/1.73mm^2^) | 118.9 ± 39.8 | 124.2 ± 37.8 | 0.433 |
| UA(μmol/L) | 356.1 ± 76.9 | 341.5 ± 95.8 | 0.373 |
| hs-CRP (mg/L) | 1.3 (0.6, 2.0) | 1.3 (0.7, 2.3) | 0.997 |
| HCY (μmol/L) | 10.6 ± 2.2 | 10.5 ± 3.5 | 0.783 |
| **Administered drugs** | | | |
| insulin, n (%) | 10 (21.3) | 22 (14.4) | 0.368 |
| OADs, n (%) | 28 (59.6) | 82 (53.9) | 0.610 |
| statins, n (%) | 4 (8.5) | 1 (0.7) | 0.013 |
| aspirin, n (%) | 2 (4.3) | 1 (0.7) | 0.275 |
| ACEI/ARB, n (%) | 10 (21.3) | 10 (6.6) | 0.008 |
| CCB, n (%) | 8 (17.0) | 16 (10.5) | 0.348 |
| β-blockers, n (%) | 3 (6.4) | 4 (2.6) | 0.443 |
| diuretic, n (%) | 0 (0.0) | 2 (1.3) | 1.000 |

Abbreviation: PAD=peripheral artery disease; WC=waist circumference; BMI=body mass index; SBP=systolic blood pressure; DBP=diastolic blood pressure; DR=diabetic retinopathy; DPN=diabetic peripheral neuropathy; DN=diabetic nephropathy; NAFLD=Nonalcoholic fatty liver disease; FBG=Fasting blood glucose; 2h PBG=2 hours postprandial blood glucose; HbA1c=glycosylated hemoglobin; HOMA-IR= homeostatic model assessment-insulin resistance; TG=triglyceride; TC=total cholesterol; HDL-C=high density lipoprotein cholesterol; LDL-C=low density lipoprotein cholesterol; eGFR=estimated glomerular filtrationrate; UA= uric acid; hs-CRP=hypersensitive C-reactive protein; HCY=homocysteine; OADs=oral antidiabetic drugs; ACEI/ARB= angiotensin-converting enzyme inhibitor/angiotensin receptor blocker; CCB=calcium channel blocker.

**Supplementary Table 2. Logistic regression analysis for PAD in patients without carotid plaque**

|  | | Univariable |  | Multivariable |  |
| --- | --- | --- | --- | --- | --- |
|  | OR(95%CI) | | p-value | aOR(95%CI) | p-value |
| legumain | 1.05(1.02-1.10) | | 0.012 | 1.05(1.02-1.10) | 0.015 |
| age | 1.04(1.01-1.08) | | 0.026 | 1.05(1.01-1.10) | 0.014 |
| male | 1.62(0.82-3.30) | | 0.173 | 1.10(0.44-2.75) | 0.833 |
| hypertension | 2.23(1.14-4.37) | | 0.019 | 1.98(0.94-4.18) | 0.072 |
| smoking history | 2.07(1.05-4.06) | | 0.035 | 2.14(0.87-5.50) | 0.103 |
| duration of diabetes | 1.04(0.98-1.10) | | 0.218 |  |  |
| LDL-C | 0.84(0.58-1.19) | | 0.325 |  |  |
| hs-CRP | 1.04(0.96-1.12) | | 0.304 |  |  |
| HCY | 1.01(0.91-1.12) | | 0.781 |  |  |
| HOMA-IR | 1.00(0.99-1.02) | | 0.638 |  |  |
| eGFR | 1.00(0.99-1.01) | | 0.431 |  |  |
| DR | 1.02(0.41-2.21) | | 0.968 |  |  |
| BMI | 0.99(0.90-1.09) | | 0.885 |  |  |
| WC | 1.01(0.98-1.05) | | 0.544 |  |  |
| SBP | 1.02(1.01-1.04) | | 0.010 |  |  |
| DPN | 0.75(0.35-1.55) | | 0.436 |  |  |
| DN | 1.17(0.57-2.22) | | 0.650 |  |  |
| stroke | 10.36(1.29-212.4) | | 0.045 |  |  |
| FBG | 1.00(0.90-1.10) | | 0.958 |  |  |
| 2hPBG | 0.99(0.92-1.06) | | 0.736 |  |  |
| HbA1C | 1.01(0.87-1.16) | | 0.920 |  |  |
| TG | 1.10(0.91-1.32) | | 0.283 |  |  |
| TC | 0.88(0.65-1.19) | | 0.412 |  |  |
| HDL-C | 1.12(0.42-2.83) | | 0.809 |  |  |
| UA | 1.00(1.00-1.01) | | 0.372 |  |  |
| insulin | 1.61(0.68-3.63) | | 0.262 |  |  |
| OADs | 1.26(0.65-2.47) | | 0.498 |  |  |
| statins | 14.14(2.03-280.51) | | 0.019 |  |  |
| aspirin | 6.76(0.63-147.35) | | 0.122 |  |  |

Abbreviation as in Supplementary Table 1.

**Supplementary Table 3. Missing values of baseline variables in total patients**

| **Characteristic** | **Missing Number (Percentage)** |  |  |
| --- | --- | --- | --- |
| **Demographic characteristics** | | | |
| age | 0(0) |  |  |
| male | 0(0) |  |  |
| WC | 3(0.62) |  |  |
| BMI | 0(0) |  |  |
| **Medical history and Clinical condition** | | | |
| smoking history | 0(0) |  |  |
| hypertension | 0(0) |  |  |
| SBP | 0(0) |  |  |
| DBP | 0(0) |  |  |
| duration of diabetes | 0(0) |  |  |
| DR | 0(0) |  |  |
| DPN | 3(0.62) |  |  |
| DN, | 0(0) |  |  |
| stroke | 0(0) |  |  |
| NAFLD | 1(0.21) |  |  |
| **Laboratory examination** | | | |
| legumain | 0(0) |  |  |
| FBG | 0(0) |  |  |
| 2hPBG | 3(0.62) |  |  |
| HbA1C | 0(0) |  |  |
| HOMA-IR | 0(0) |  |  |
| TG | 0(0) |  |  |
| TC | 0(0) |  |  |
| HDL-C | 0(0) |  |  |
| LDL-C | 0(0) |  |  |
| eGFR | 0(0) |  |  |
| UA | 0(0) |  |  |
| hs-CRP | 3(0.62) |  |  |
| HCY | 1(0.21) |  |  |
| **Administered drugs** | | | |
| insulin | 0(0) |  |  |
| OADs | 0(0) |  |  |
| statins | 0(0) |  |  |
| aspirin | 0(0) |  |  |
| ACEI/ARB | 0(0) |  |  |
| CCB | 0(0) |  |  |
| β-blockers | 0(0) |  |  |
| diuretic | 0(0) |  |  |

Abbreviation as in Supplementary Table 1.
